# Genetic variants in the upstream region of activin receptor IIA are associated with female fertility in Japanese Black cattle

**DOI:** 10.1186/s12863-015-0282-0

**Published:** 2015-10-20

**Authors:** Shinji Sasaki, Takayuki Ibi, Tamako Matsuhashi, Kenji Takeda, Shogo Ikeda, Mayumi Sugimoto, Yoshikazu Sugimoto

**Affiliations:** National Livestock Breeding Center, Odakura, Nishigo, Fukushima 961-8511 Japan; Graduate School of Environmental and Life Science, Okayama University, Tsushima-naka, Okayama 700-8530 Japan; Gifu Prefectural Livestock Research Institute, Kiyomi, Takayama, Gifu 506-0101 Japan; Cattle Breeding Development Institute of Kagoshima Prefecture, Osumi, So, Kagoshima, 899-8212 Japan; Shirakawa Institute of Animal Genetics, Japan Livestock Technology Association, Odakura, Nishigo, Fukushima 961-8061 Japan

**Keywords:** Female fertility, Reproductive efficiency, Genome-wide association study, Activin receptor IIA (*ACVR2A*), Japanese Black cattle, Beef cattle

## Abstract

**Background:**

Female fertility, a fundamental trait required for animal reproduction, has gradually declined in the last 2 decades in Japanese Black cattle. To identify associated genetic variants in Japanese Black cattle, we evaluated female fertility as a metric to describe the average inverse of the number of artificial inseminations required for conception from the first through the fourth parity (ANAI_4_) and conducted a genome-wide association study (GWAS) using 430 animals with extreme ANAI_4_ values from 10,399 animals.

**Results:**

We found that 2 variants, namely a single-nucleotide polymorphisms (SNP; g.48476925C > T) and a 3-bp indel (g.48476943_48476946insGGC), in the upstream region of the activin receptor IIA gene (*ACVR2A*) were associated with ANAI_4_. *ACVR2A* transcripts from Japanese Black cattle of the *Q* haplotype, defined by the SNP and the 3-bp indel, with increased ANAI_4_ were 1.29–1.32-fold more abundant than *q*-derived transcripts. In agreement, reporter assay results revealed that the activity of the *ACVR2A* promoter was higher in reporter constructs with the *Q* haplotype than in those with the *q* haplotype by approximately 1.2 fold. Expression of exogenous *ACVR2A* induced dose-dependent increases of reporter activity from the follicle-stimulating hormone, beta polypeptide (*FSHB*) promoter in response to activin A in a pituitary gonadotrophic cell line. The findings suggested that sequence variations in the upstream region of *ACVR2A* with the *Q* haplotype increased *ACVR2A* transcription, which in turn induced *FSHB* expression*.* This association was replicated using a sample population size of 1,433 animals; the frequency of the *Q* haplotype was 0.39, and *Q*-to-*q* haplotype substitution resulted in an increase of 0.02 in terms of ANAI_4_.

**Conclusions:**

This GWAS identified variants in the upstream region of *ACVR2A*, which were associated with female fertility in Japanese Black cattle. The variants affected the level of *ACVR2A* mRNA expression, which could lead to an allelic imbalance. This association was replicated with a sample population of 1,433 animals. Thus, the results suggest that the *Q* haplotype could serve as a useful marker to select Japanese Black cattle with superior female fertility.

**Electronic supplementary material:**

The online version of this article (doi:10.1186/s12863-015-0282-0) contains supplementary material, which is available to authorized users.

## Background

Fertile female cattle show clear estrous in a timely manner and become pregnant within a minimum number of artificial inseminations (AIs). However, over the past 2 decades, female fertility in AI breeding programs has been gradually declining in Japanese Black cattle; e.g., the first-AI conception rate decreased from 67.4 % to 56 % between 1992 and 2012 in Japan [1]. The trend has been also observed in dairy cattle [1, 2]. Additional AIs increase costs related to semen, hormonal treatments, and AI technician fees, as well as feeding until the next AI. Therefore, farmers and breeders pay close attention to genetic factors related to improving female fertility for greater reproductive performance and profitability.

Recently, using a high-density single-nucleotide polymorphism (SNP) array [3, 4], genome-wide association studies (GWAS) have enabled researchers to scan the entire genome for related genetic factors and have identified a quantitative trait locus (QTL) for fertility-related traits in various cattle breeds [5–13] (reviewed in [14, 15]); however, QTLs for fertility-related traits in Japanese Black cattle have remained fully unknown.

Japanese Black cattle are highly valued owing to the abundant marbling of meat caused by intramuscular fat depositions [16]. Strict selection for marbling under a closed breeding system in Japan [17] has made Japanese Black cattle genetically distinct from European cattle breeds [18]. Therefore, genome-wide QTL screening for female fertility needs to be applied to this breed.

The genetic parameters for calving interval-related traits have been evaluated until animals reach approximately 4 to 5 years of age in Japanese Black cattle in Japan [19]. Thus, the AI records of 10,399 animals at each parity, from the first through the third or fourth parity (see Methods section) were available. Multiple records from single animals are important for accurately evaluating fertility performances. However, the number of cows decreases as the age of cows increases, reflecting the culling of animals with lower fertility. Thus, in this study, to identify variants associated with female fertility in Japanese Black cattle, we evaluated female fertility as a metric to describe the average inverse of the number of AIs required for conception [20] from first through fourth parity (ANAI_4_) and conducted a GWAS for this trait. The current GWAS identified associated variants in the upstream region of the activin receptor IIA gene (*ACVR2A*), which serve as a key regulator of follicular growth in the ovaries by controlling follicle-stimulating hormone (*FSH*) expression.

## Results and discussion

### A QTL for ANAI_4_ was identified on bovine chromosome 2 (BTA2) in Japanese Black cattle

**Fig. 1:**
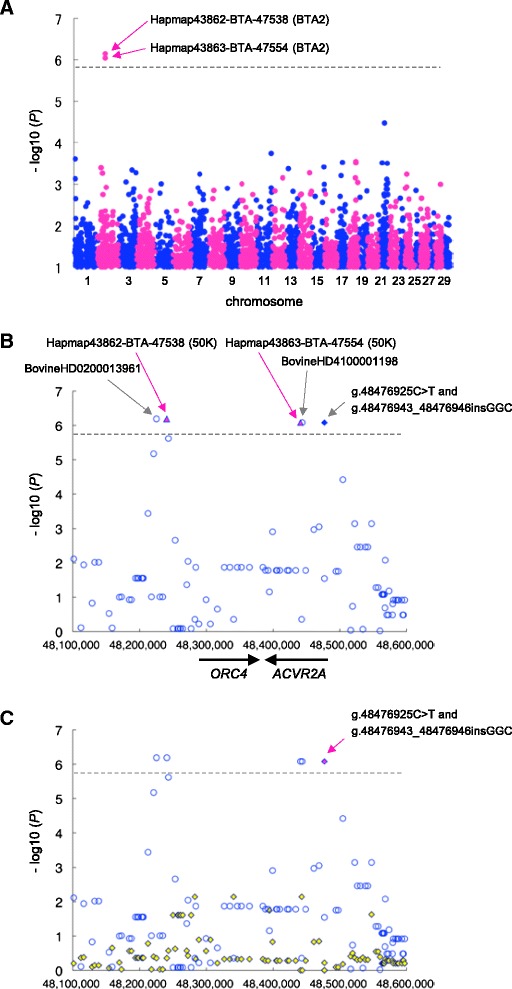
Association of SNPs and a indel with ANAI_4_ in 430 Japanese Black cattle. **a** Manhattan plot of the association of 33,303 SNPs (BovineSNP50K BeadChip) with ANAI_4_ in 430 Japanese Black cattle. The chromosomes are distinguished with alternating colors (blue, odd numbers; red, even numbers). The chromosome number is indicated on the X-axis. The dashed line is the Bonferroni-corrected threshold for genome-wide significance (−log10 (*P*) = 5.823). The vertical axis is broken for *P* values below -log10 (*P*) = 1. **b** Regional plot of the locus on BTA2 associated with ANAI_4_. SNPs from the BovineSNP50K BeadChip are shown as red triangles. The imputed SNPs are shown as unfilled blue circles. g.48476925C > T SNP and the g.48476943_48476946insGGC indel are shown as filled blue diamonds. Genes and their directions of transcription are noted at the bottom of the plot. **c** A conditioned analysis was performed by including the haplotype, defined by g.48476925C > T SNP and the g.48476943_48476946insGGC indel, as covariates in the model*.* The 2 red, filled diamonds indicate the g.48476925C > T and g.48476943_48476946insGGC (arrow) variants. The blue, unfilled circles and the yellow, filled diamonds represent *P* values on a –log10 scale before and after conditioning, respectively. The positions shown are based on the UMD3.1 assembly of the bovine genome

The heritability of ANAI_4_ was estimated to be 0.02 using the numerator-relationship matrix among 10,399 animals based on pedigree information, consistent with previous studies reporting the inverse of the number of inseminations required for conception [20] and female conception-related traits in cattle (reviewed in [15, 21]). As shown in Additional file 1, the distribution was sufficiently wide to discriminate between the upper- and lower-performance groups. Selective genotyping using animals with phenotypic values that deviate from the population mean is an effective method for reducing the sample sizes required to detect common SNPs associated with traits [22]. We selected 256 cows from the upper extreme (85^th^ percentile, average ANAI_4_ was 0.927) of the distribution and 174 cows from the lower extreme (15^th^ percentile, average ANAI_4_ is 0.399) among 10,399 cows with ANAI_4_ records. These samples were genotyped using the BovineSNP50K BeadChip, comprising probes for 54,001 SNPs. A total of 33,303 autosomal SNPs that passed our quality control criteria (call rate > 99 %, minor allele frequency > 0.01, Hardy–Weinberg equilibrium *P* > 0.001, and inclusion of the SNP on the BovineHD BeadChip) [23] were used for the association study. Analysis was performed using EMMAX software [24], which is based on a linear-mixed model approach using a genetic-relationship matrix estimated by SNP genotypes to model the correlation between the phenotypes of the sample subjects. The genomic-inflation factor (λ GC) in this analysis was 1.058, indicating that the sample was appropriate for an association study. A quantile–quantile (Q–Q) plot showed that 2 SNPs deviated from the distribution under the null hypothesis (Additional file 2). Two SNPs on BTA2 reached the Bonferroni-corrected threshold for genome-wide significance (*P* < 1.5 × 10^−6^; Fig. 1a, Table 1). The 2 SNPs, Hapmap43862-BTA-47538 and Hapmap43863-BTA-47554, were located within a 200-kbp window from 48,240,577 bp to 48,440,885 bp on BTA2 and were in linkage disequilibrium (LD) with each other (*P* < 6.52 × 10^−7^–8.32 × 10^−7^, *D′* =1, *r*^2^ = 0.99), as shown in Table 1. This QTL has not been previously reported for reproductive-related traits in cattle (reviewed in [14, 15]; for a cattle QTLdb, see [25]).

**Table 1:** SNPs and a indel with genome-wide significant associations with ANAI_4_ on BTA2

BTA	SNP and indel-ID	Reference SNP-ID_number^c^	position (bp) _UMD3.1^d^	allele_1^e^	Minor allele frequency (upper extrame)	Minor allele frequency (lower extrame)	allele_2^f^	odds ratio	P-value
2	BovineHD0200013961^b^	rs110523739	48225372	G	0.45	0.29	A	1.98	6.52E-07
2	Hapmap43862-BTA-47538^a^	rs41636186	48240577	A	0.45	0.29	G	1.98	6.52E-07
2	Hapmap43863-BTA-47554^a^	rs41636197	48440885	G	0.45	0.29	A	1.96	8.32E-07
2	BovineHD4100001198^b^	rs41636199	48443632	A	0.45	0.29	G	1.96	8.32E-07
2	g.48476925C>T		48476925	T	0.45	0.29	C	1.96	8.32E-07
2	g.48476943_48476946insGGC		48476943_48476946	GGC	0.45	0.29	(-)	1.96	8.32E-07

^a^SNPs included in the Illumina Bovine SNP50K BeadChip; ^b^Imputed SNPs from the Illumina Bovine HD BeadChip. ^c^Reference SNP ID numbers (rs) were obtained using the SNPchiMp v.3 database (http://bioinformatics.tecnoparco.org/SNPchimp/). ^d^The positions are based on the UMD3.1 assembly of the bovine genome. ^e^Minor allele; ^f^Major allele. The upper and lower extremes correspond to ANAI_4_ values above the 85th percentile and below the 15th percentile, respectively

To characterize the region on BTA2 in more detail—in particular, the extent of LD in the QTL region, the genotypes of 430 animals for 33,303 SNPs were imputed using BEAGLE software [26, 27] with phased haplotype data inferred from 586,812 SNPs (BovineHD Beadchip) in 1,041 Japanese Black cattle served as the reference [23]. We previously estimated the imputation accuracy by comparing the true genotypes to the imputed genotypes, indicating that such imputation was highly accurate [23]. Subsequently, 4 SNPs associated with ANAI_4_ were detected within the 218-kbp from 48,225,372 bp to 48,443,632 bp on BTA2 using EMMAX (*D′* = 1, *r*^2^ ranging from 0.99 to 1.00; Fig. 1b, Table 1). Except for the 4 SNPs on BTA2, we did not detect any imputed SNPs that were significantly associated with ANAI_4_ (*P* < 0.05, Bonferroni-corrected).

### Variants in the upstream region of *ACVR2A* were associated with ANAI_4_

The LD region harbors 2 genes: origin recognition complex subunit 4 (*ORC4*) and *ACVR2A* (Fig. 1b, Additional file 3). Out of 4 associated SNPs, 2 SNPs were located in the intronic region of *ACVR2A* and 2 SNPs were located on centromeric side at a distance of 42 to 57 kbp from *ORC4*, respectively (Fig. 1b). To detect associated polymorphisms in *ORC4* and *ACVR2A*, we sequenced all exons and upstream regions, beginning 2,131 bp upstream of the start codon, of each gene in 3 animals with homozygous *Q* and *q* haplotypes that were defined by the genotypes of Hapmap43862-BTA-47538 (48,240,577 bp) and Hapmap43863-BTA-47554 (48,440,885 bp) (Fig. 1a, B, Table 1). In the region of *ACVR2A*, we found a 21-bp indel (g.48476691_484766711delGAGCTCGCGGCGGTGGCGGCC) in the 5′-untranslated region (UTR) and a SNP in 3′-UTR (g.48381943A > G) in exons 1 and 11, respectively. We also found a SNP (g.48476925C > T) and a 3-bp indel (g.48476943_48476946insGGC) in the upstream region of *ACVR2A* (716 bp and 737–740 bp upstream of the start codon, respectively). We did not found any variants in the exons or in the region upstream of the *ORC4* transcription start site. To ascertain whether the *ACVR2A* variants were associated with ANAI_4_, we genotyped the variants in 430 animals used in the GWAS and analyzed the association with ANAI_4_, using EMMAX software and a genetic-relationship matrix for the animals. The SNP (g.48476925C > T) and the 3-bp indel (g.48476943_48476946insGGC) in the upstream region of *ACVR2A* produced a highly significant signal (*P* = 8.32 × 10^−7^; Fig. 1b, Table 1), whereas the 21-bp indel in the 5′-UTR and the SNP in the 3′-UTR of *ACVR2A* were not associated with ANAI_4_ (*P* = 0.028 and *P* = 0.51, respectively). Subsequently, 5 SNPs and 1 indel associated with ANAI_4_ were detected within the 251.5-kbp region spanning 48,225,372 bp to 48,476,946 bp on BTA2 (*D′* =1, *r*^2^ ranging from 0.99 to 1.00; Fig. 1b, Table 1).

We then performed conditioned analysis to ascertain whether there were any other significantly associated SNPs in the region. The *Q* haplotype, defined by the SNP (g.48476925C > T) and the 3-bp indel (g.48476943_48476946insGGC) in the upstream region of *ACVR2A*, was individually included as a covariate in the linear-mixed model. After conditioning, the associations of other SNPs were no longer evident (Fig. 1c), indicating that the region contained a single QTL.

ACVR2A is a type-II transforming growth factor-beta (TGF-beta) receptor with serine/threonine kinase activity, which is involved in initial activin binding. Such binding leads to the recruitment and phosphorylation of type-I TGF-beta receptors and activates transcription of specific target genes, such as the FSH, polypeptide beta gene (*FSHB*) (reviewed in [28]). FSH is a heterodimer, consisting of an alpha and beta subunit. FSH is produced in gonadotropes in the anterior pituitary gland in response to activin by autocrine and paracrine mechanisms, and stimulates the growth and recruitment of immature follicles in the ovary [29]. Given these facts, *ACVR2A* could be a reasonable candidate gene for the fertility trait.

### Variants in the 5′-upstream region of *ACVR2A* were involved in allelic imbalances of *ACVR2A* mRNA expression

**Fig. 2:**
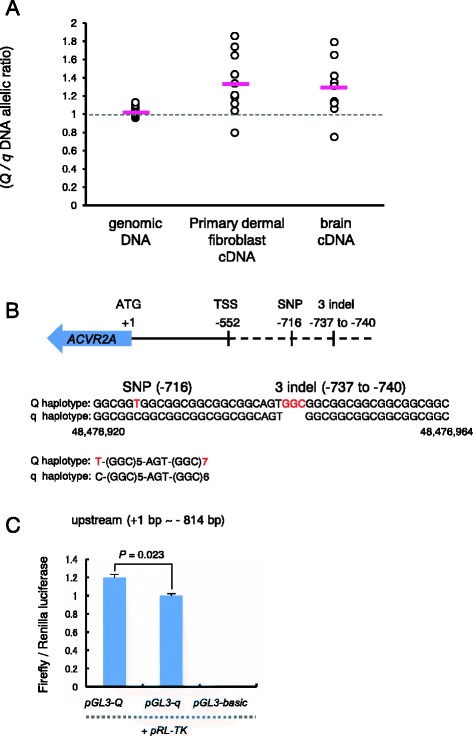
The upstream region of *ACVR2A* affects *ACVR2A* expression. **a** Allelic-imbalance test for the level of *ACVR2A* mRNA expression in heterozygotes. cDNA from primary dermal fibroblasts and brain, and gDNA from heterozygous animals were amplified using primers to BovineHD4100001198 (48,443,632 bp), which is located in an intron of *ACVR2A* (Table 1). The PCR product was directly sequenced. Peak heights at the SNP site were quantified using PeakPicker 2 software [30]. The Y-axis shows the ratio of the peak height of the *Q* allele to that of the *q* allele in gDNA (*n* = 16, mean = 1.035), cDNA from primary dermal fibroblasts (*n* = 13, mean = 1.32), or cDNA from brains (*n* = 9, mean = 1.29). Red bars show the mean. The *P* values for the difference between cDNA and gDNA were 0.009 in primary dermal fibroblasts and 0.044 in brain, respectively, as determined by performing *t* tests. **b** Schematic representation of the positions of variants in the 5′ upstream region of *ACVR2A*. “SNP (−716)” and “3 indel (−737 to −740)” represent g.48476925C > T and g.48476943_48476946insGGC, respectively. “ATG (+1)” and “TSS (−522)” represent the start codon and the predicted transcriptional start site, respectively. A *Q* and *q* haplotype sequence alignment of the upstream region of *ACVR2A* encompassing 48,476,920 and 48,476,964 bp is shown. **c** Luciferase reporter assays for the 5′ upstream region of *ACVR2A*-derived *Q* and *q* haplotypes. The 5′ upstream region (814 bp upstream from the start codon) derived from the *Q* and *q* haplotypes was cloned into the firefly luciferase pGL3-*Q* and pGL3-*q* plasmids, respectively. The Firefly-to-Renilla luminescence ratios observed after cotransfecting HeLa cells were measured to evaluate the effects of the 5′ upstream region. Bars represent the mean ± SEM obtained in triplicate from 3 independent experiments. *P* values determined by *t* tests are shown

Variants in the upstream region of the *ACVR2A* gene could potentially affect the activity of the promoter and, thus, they may contribute to an allelic imbalance in *ACVR2A* mRNA expression. To compare the relative abundance of *Q*- versus *q*-derived transcripts of *ACVR2A*, we performed allelic-imbalance testing with heterozygous samples [30]. The exonic SNPs in 5′ and 3′-UTRs of *ACVR2A* were not associated with ANAI_4_. The intronic SNPs were transcribed as primary mRNAs in the nucleus; thus, we amplified an intronic *ACVR2A* SNP, BovineHD4100001198 (48,443,632 bp, Table 1), which is in perfect LD with the *Q* haplotype that was defined by the g.48476925C > T SNP and the 3-bp g.48476943_48476946insGGC indel genotypes (*r*^2^ = 1). We observed that *ACVR2A* was ubiquitously expressed in female cow tissues including primary dermal fibroblasts and brain tissues (Additional file 4). Therefore, we compared the relative abundances of *Q*- versus *q*-derived *ACVR2A* transcripts in primary dermal fibroblast (*n* = 13) and brain tissues (*n* = 9) from heterozygotes (Fig. 2a). We isolated samples of genomic DNA (gDNA) and complementary DNA (cDNA) derived from total RNA of whole-cell lysates and then compared their allelic ratios using PeakPicker2 software [30]. The results showed that the *Q*-derived *ACVR2A* transcripts were 1.32-fold and 1.29-fold more abundant than *q*-derived transcripts in primary dermal fibroblasts and in brain (*t*-test, *P* = 0.009 and *P* = 0.044, respectively). In contrast, *Q*-derived *ACVR2A* gDNA was detected equally well as *q*-derived gDNA (Fig. 2a).

To determine whether the imbalance in the *ACVR2A* transcript ratio between the haplotypes was attributable to the variants in the upstream region, we cloned the upstream region, beginning 814-bp upstream of the start codon, which included the g.48476925C > T SNP and the 3-bp indel g.48476943_48476946insGGC from both the *Q* and *q* haplotypes, into luciferase-reporter constructs (Fig. 2b). We then transfected HeLa cells with these constructs and measured the resulting luciferase activities at 24 h post-transfection. The activity was higher for the *Q* constructs than for the *q* constructs, with a difference of approximately 1.2 fold (*t-*test, *P* = 0.023) (Fig. 2c). These results suggest that the variants including the SNP (g.48476925C > T) and the 3-bp indel (g.48476943_48476946insGGC) in the upstream region of *ACVR2A* affected the level of *ACVR2A* mRNA expression, which could lead to an allelic imbalance in *ACVR2A* mRNA expression.

We did not observe that the SNP (g.48476925C > T) or the 3-bp indel (g.48476943_48476946insGGC) resided in a transcription factor-binding site in the upstream region of *ACVR2A* in either the *Q* or *q* haplotypes, using the TRANSFAC Professional database [31]. The SNP (g.48476925C > T) is a T(*Q*) to C(*q*) SNP located 716 bp upstream of the start codon of *ACVR2A*, and the 3-bp indel (g.48476943_48476946insGGC) is a (GGC)n trinucleotide repeat with either 7(*Q*) or 6 (*q*) copies located 737–740 bp upstream of the start codon of *ACVR2A* (Fig. 2b). Recently, Karim et al. reported that 2 causative variants for bovine stature QTL in the upstream region of *PLAG1* influence the promoter activity and reflect differential binding of nuclear factors [32]. The causative variants were a (CCG)n trinucleotide repeat with either 11(*Q*) or 9(*q*) copies located immediately upstream of the presumed *PLAG1* transcription start site and a G(*Q*) to A(*q*) SNP located 12-bp upstream of the (CCG)n trinucleotide repeat [32]. The repetitive, GC-rich triplet composition and the location were similar to that observed in the upstream region of *ACVR2A* (Fig. 2b). Therefore, it is possible that the variants in the upstream region of *ACVR2A* could also affect promoter activity and cause differential binding of transcriptional factors between the *Q* and *q* haplotype, which in turn may have caused the imbalance observed in *ACVR2A* transcription between animals with *Q* and *q* haplotypes.

### *FSHB* expression is dependent on *ACVR2A* expression in a gonadotrope cell line

Matzuk et al. [31] found that female *Acvr2a-*knockout mice were infertile and showed evidence of multiple follicle atresias in the ovaries. Consistently, FSHB expression in the anterior pituitary was suppressed in *Acvr2a-*knockout mice, and serum FSH was decreased in female homozygous *Acvr2a-*knockout mice (35.2 ± 5.2 ng/ml) compared to female wild-type mice (83.4 ± 20.6 ng/ml), indicating that ACVR2A affects folliculogenesis by regulating *FSHB* expression. However, it is unknown whether the level of ACVR2A influences *FSHB* expression in a dose-dependent manner.

The mouse gonadotrope LβT2 cell line [33, 34] shows *FSHB* expression and FSH secretion that is induced in response to activin A through ACVR2A [35–37]. Therefore, this cell type serves as a good model for analyzing whether the level of *ACVR2A* expression influences *FSHB* expression, using an *FSHB* promoter-reporter plasmid [36]. Although the transfection efficiency in LβT2 cells was very low (8.8 ± 1.62 % of 3,368 cells; Additional file 5: Figure S5A) [36], the co-transfection efficiency was approximately 100 % (100 % of 3,368 cells; Additional file 5: Figure S5B–D). These findings indicated that *FSHB* promoter-reporter activity was arguably detected in cells co-transfected cells with the *ACVR2A* expression plasmid. In addition, we confirmed that the level of ACVR2A was dependent on amount of the *ACVR2A* expression plasmid used for the transfections (Additional file 6: Figure S6).

**Fig. 3:**
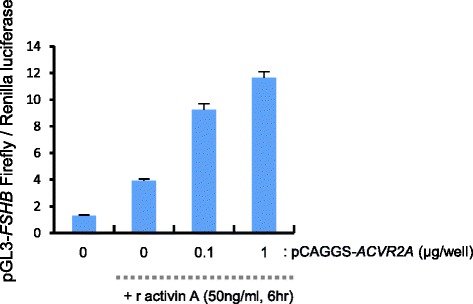
*FSHB* promoter activity is dependent on *ACVR2A* expression in response to activin A in LβT2 cells. The pGL3-Basic-*FSHB* (chr2:107,059,651–107,061,641 on the GRCm38/mm10 mouse genome assembly) and pCAGGS-*ACVR2A* plasmids were cotransfected with pRL-TK into LβT2 cells. At 18 h post-transfection, recombinant activin A was added for 6 h, and the Firefly-to-Renilla luminescence ratios were measured. Bars represent the mean luminescence ± SEM obtained in triplicate from 3 independent experiments. *P* values were determined by Tukey–Kramer post-hoc test (*P* < 0.01)

To determine whether the level of *ACVR2A* affects *FSHB* expression in response to activin A, we co-transfected LβT2 cells with an *ACVR2A*-expression plasmid and an *FSHB* promoter-reporter plasmid. The results showed that activin A-induced *FSHB* promoter activity was dependent on the amount of *ACVR2A* expression plasmid used (Fig. 3; Tukey–Kramer post-hoc test, *P* < 0.01). These finding suggested that the level of *ACVR2A* affected the level of *FSHB* expression in response to activin A.

Taken together, these results suggested that the variants in the upstream region of *ACVR2A* in the *Q*-haplotype animals induced an increase in *ACVR2A* transcription relative to that observed in the *q* animals, which in turn could induce *FSHB* expression. Cattle are polyestrous animals and display estrous behavior approximately every 21 days. Normally, 2–3 waves of follicular growth occur in the ovaries during each estrous cycle, which are induced by a transient increase of FSH concentration [38–40]. Subsequently, a single dominant follicle is selected in a manner that is dependent on decline of the FSH concentration [41], followed by ovulation of the single dominant follicle or atresia. Although cattle are mono-ovulatory species, FSH concentrations influence the emergence of co-dominant follicles [42]. Moreover, administration of exogenous recombinant FSH in cattle induces multiple follicles growth during super-ovulation [43]. In this manner, follicular growth at several stages is closely associated with circulating FSH concentrations. Therefore, although quantitative differences in FSHB expression between *Q-* and *q*-haplotype animals may be subtle, it is possible that these differences govern folliculogenesis. Thus, further analyses regarding differences of serum FSH levels and follicle size at each stage between *Q* and *q*-haplotype animals could help to elucidate the mechanisms underlying the variants of *ACVR2A* that influence female fertility.

Of note, mutation of TGF-beta family ligand and its receptor BMP15 [44] and the type-I receptor ALK6 [45, 46] were identified as causative variants in the ovulation rate in sheep. Inactivation of 1 copy of *BMP15* increased the ovulation rate, whereas inactivation of both copies of *BMP15* showed blocked follicular growth. In contrast, *ALK6* mutant alleles increased the ovulation rate in an additive manner, indicating that the TGF-beta family ligand and its receptor functions in folliculogenesis in a dosage-sensitive manner. Similarly, ACVR2A, which is a TGF-beta family type II receptor, may also function in female fertility in cattle in a dose-dependent manner.

### Replication of the associated haplotype in a sample of 1,433 animals

To validate the GWAS results and estimate the effective size of the QTL and the allele frequencies, we examined whether the *Q* haplotype was associated with ANAI_4_ in a sample of 1,433 animals. We genotyped 1,433 animals randomly selected from the remainder of the cohort from the same farm used for the GWAS (Additional file 7 ). The results showed that the *Q* haplotype was significantly associated with ANAI_4_ compared to the *q* haplotype (Tukey–Kramer post-hoc test, *P* = 0.017; Table 2). The *Q* haplotype frequency was 0.39, indicating that the haplotype is common in Japanese Black cattle. We fitted a linear-mixed model to the ANAI_4_ values in the additive model and used restricted maximum likelihood (REML) analysis to estimate the variance explained by the haplotype. We estimated the proportion of total genetic variance attributable to the *Q* haplotype as 0.1 (Table 2). The *Q*-to-*q* haplotype substitution effect on ANAI_4_ was 0.02 (Table 2).

**Table 2:** Proportion of genetic variance attributable to haplotypes associated with ANAI_4_

BTA	BTA SNP and indel-id	positon (bp) _UMD3,1^a^	*Q/q* haplotype	Number of animals genotyped for the haplotype	Minor allele frequency	Heritability	Haplotype effect on total genetic variance^b^	Haplotype substitution P-value^d^ effect^c^	P-value^d^
2	g. 48476925C>T and g.48476943_48476946insGGC	48476925 and 48476943. 48476946	T and GCC/C and (-)	1433	0.39	0.02	0.1	0.02	0.017

^a^The positions shown are based on the UMD3.1 assembly of the bovine genome.

^b^The effects of the haplotype were estimated as the least-square means from GLM analysis. The statistical model for GLM analysis included the fixed variables of the farm, birth year, and haplotype. The genetic variance explained by the haplotype was calculated based on estimates of the haplotype effect and the haplotype frequency [55]. Total genetic variance was estimated using the MTDF-REML programs. The effect size of haplotype was estimated as the proportion of genetic variance explained by the haplotype.

^c^The average ANAI_4_ values for *QQ* and *qq* were 0.69 and 0.65, respectively.

^d^Results were tested by a 1-way ANOVA, followed by the Tukey–Kramer test for multiple comparisons

## Conclusion

This GWAS identified variants in the upstream region of *ACVR2A*, which were associated with female fertility in Japanese Black cattle. The variants affected the level of *ACVR2A* mRNA expression, which led to an allelic imbalance. Expression of exogenous *ACVR2A* induced dose-dependent increases of *FSHB* expression in response to activin A. Finally, we replicated this association and estimated the effect in a sample of 1,433 animals. Thus, the results suggest that *Q* haplotype could serve as a useful marker in Japanese Black cattle to select animals with superior female fertility in Japanese Black cattle.

## Methods

### Ethics statement

All animal experiments were performed according to the guidelines for care and use of laboratory animals of Shirakawa Institute of Animal Genetics, and this research project was approved by the Shirakawa Institute of Animal Genetics Committee on Animal Research (H21-1). We have obtained the written agreement from the cattle owners to use the samples and data.

### Collection of phenotype data

Data were collected from cattle farms, and the data management system for Japanese Black cattle was described in a previous study [47, 48]. The original data included 63,775 records for reproductive females born from 1992 to 2006. The data were selected using 9 selection criteria: 1) data were not missing for the cow from first to fourth parity, 2) the cow did not have twins in parturition, 3) the cow did not receive any embryo transfers, 4) the cow did not have any abortions, 5) the length of all gestations ranged from 261 to 310 days, 6) the calving interval ranged from 276 to 730 days, 7) the age of the cow at the first calving was be less than 1,128 days, 8) the cow was reared in a single farm, and 9) each breeding farm had more than 10 records from each birth year. After applying these selection criteria, the final dataset contained 10,399 records. In this study, female fertility was evaluated as a metric to describe the average inverse of the number of artificial inseminations required for conception [20] from the first parity through the fourth parity. For example, if conception is achieved at the first, second, third, or fourth AI, ANAI values are 1, 0.5, 0.33, and 0.25, respectively. ANAI_4_ values were corrected for the effects of the individual farm and birth year. The heritability and variance components of phenotypic variance were estimated with the REML procedure using the MTDF-REML programs [49]. We fitted single-traits animal models with random effects and fixed effects:

$$ {y}_{ij}=\mu +yea{r}_i+ far{m}_j+{u}_{ij}+{e}_{ij} $$

where *y*_*ij*_ is the observation of *ij* for the traits, μ is the total mean, *year*_*i*_ is the fixed effect of birth year *i* (15 classes, 1992 to 2006), *farm*_*j*_ is the fixed effect of farm *j* (174 classes), and *u*_*ij*_ is the infinitesimal genetic effect of animal *ij*, which is distributed as *N*(0, **A***σ*_*u*_^2^), where **A** is the numerator relationship matrix, and *e*_*ij*_ is the residual effect. Pedigrees of the base population animals were traced back for 2 generations to create the numerator relationship matrix, and 10,399 animals were included in the pedigree analysis.

### Selection of samples for the GWAS and DNA sample collection

Samples were selected from the upper extreme (85^th^ percentile, 1,560 animals) and the lower extreme (15^th^ percentile, 1,560 animals) for ANAI_4_. To reduce population stratification, we selected less than 5 cows derived from a single sire in each extreme, resulting in 256 cows for the upper extreme and 174 cows for the lower extreme. Whole blood was collected from each cow, and gDNA was isolated using the Easy-DNA gDNA Purification Kit (Invitrogen, Cat. #K1800-01).

### GWAS for ANAI_4_

A total of 430 DNA samples were genotyped using the BovineSNP50K BeadChip (version 1, Illumina), which comprises probes for 54,001 SNPs. The UMD3.1 assembly [50] was used to map the positions of the SNPs. The data were analyzed using PLINK software, v1.07 [51]. A total of 33,303 autosomal SNPs that passed our quality control criteria (call rate > 99 %, minor allele frequency > 0.01, Hardy–Weinberg equilibrium *P* > 0.001, and inclusion of the SNP on the Illumina Bovine HD BeadChip) [23] were used for the association study. We performed a GWAS using the trait as a binary variable, as is commonly done in case–control studies. To analyze the upper- and lower-performance phenotypes, we used a linear mixed model with a genetic relationship matrix for the binary phenotypes using the EMMAX software [24].

### Linkage disequilibrium and diplotype analysis

Haploview 4.2 software [52] was used to analyze linkage disequilibria between the SNPs. The diplotypes of the GWAS samples were estimated using BEAGLE3.3.2 software [26, 27].

### Imputation of SNPs

The genotypes of 33,303 SNPs were imputed using BEAGLE 3.3.2 software, with phased haplotype data inferred from 586,812 SNPs in 1,041 Japanese Black cattle as the reference [23].

### Expression analysis

For real-time quantitative PCR, we extracted total RNA from cow tissues, primary bovine dermal fibroblasts, and bovine endometrial epithelial cells (Cell Application, Inc., Cat. #B932-05) using RNeasy Mini Kits (Qiagen, Cat. #74104), after which the total RNA was treated with DNase I. cDNA was synthesized from 50 ng total RNA using the ReverTra Ace-α Kit (Toyoba, Cat. #FSK-101) with random primers, according to the manufacturer’s instructions. The *ACVR2A* gene was detected with the following primers and probe: forward, 5′-catgggattagtcctgtgggaac-3′; reverse, 5′-cctcaaatggcagcatgtattca-3′; and probe, 5′-tacaggtccatctgcagcagtacagcga-3′. Real-time PCR was performed on a 7900HT Real-Time PCR System (Applied Biosystems) using the comparative Ct method with glyceraldehyde-3-phosphate dehydrogenase mRNA serving as the internal control.

### Allelic imbalance test

To quantify the allelic imbalance of *ACVR2A* transcripts, we designed PCR primers to BovineHD4100001198 (48,443,632 bp) on BTA2, located in an intron of *ACVR2A.* The forward primer was 5′-aacctagaaaccgtagaaagacga-3′, and the reverse primer was 5′-gatggcatctcttggctcat-3′. We used 50 ng of template cDNA from primary bovine dermal fibroblasts or bovine brain tissues (medulla oblongata), or 10 ng of gDNA from heterozygous animals for PCR amplification with TaKaRa Ex Taq HS DNA Polymerase (TaKaRa, Cat. #RR006). The PCR product was directly sequenced and purified with the CleanSEQ system (Agencourt, Cat. #A29154). Peak heights at polymorphic sites were quantified using PeakPicker 2 software [30]. Allelic imbalances were estimated as the ratio of the peak height of the *Q* allele to that of the *q* allele in cDNA and in gDNA. Calibration curves were generated using data obtained by mixing varying amounts of gDNA from *Q* and *q* homozygotes.

### Luciferase reporter assays

To measure the effects of the 5′-upstream region of *ACVR2A* on *ACVR2A* expression, the 814-bp fragment upstream of the start codon of *ACVR2A* of each haplotype (*Q* and *q* haplotype) including a SNP (g.48476925C > T) and a 3-bp indel (g.48476943_48476946insGGC), was PCR amplified using PrimeSTAR Max DNA Polymerase (Takara, Cat. #R045A). PCR was performed using gDNA, a forward primer (5′-GGGGTACCacaatctcctcgcgctcac-3′; uppercase letters indicate the *Kpn*I linker), and a reverse primer (5′-TCCCCCGGGactttgcagcagctcccatt-3′; uppercase letters indicate the *Sma*I linker). The PCR products were cloned into the *Kpn*I and *Sma*I sites of the pGL3-Basic Vector (Promega, Cat. #E1751). The sequence and orientation of the insert were confirmed by sequencing. The pGL3-Basic Vector was used for mock transfections. For cell culture, HeLa S3 cells were maintained in Dulbecco’s modified Eagle’s medium (DMEM; Sigma, Cat. #D5796) with 10 % fetal calf serum (FCS; Sigma, Cat. #F-2442) supplemented with 2 mM l-glutamine (Gibco, Cat. #25030-081) and 100 units/ml penicillin and 100 μg/ml streptomycin (Gibco, Cat. #15140-122). Using Lipofectamine 2000 (Invitrogen, Cat. #11668-019), we transfected 5 × 10^4^ cells per well in a 24-well plate with a mixture of 750 ng of the reporter vector and 10 ng of the pRL-TK Renilla vector (Promega, Cat. #E2241) to calibrate transfection efficiency. Luciferase assays were performed 24 h post-transfection using the Dual Luciferase Reporter Assay System (Promega, Cat. #E1910) and the GloMax (Promega, Cat. #E6521).

### *FSHB* promoter-reporter assay

To measure the *FSHB* promoter activity, the promoter region of *FSHB* (chr2:107,059,651–107,061,641 on the GRCm38/mm10 mouse genome assembly) [36] was PCR amplified from gDNA of a C57BL/6NJ mouse using a forward primer (5′-GGGGTACCcctgttcattaaccactgagct-3′; uppercase letters indicate the *Kpn*I linker) and a reverse primer (5′-CCGCTCGAGcactgagtcaagttacacctca-3′; uppercase letters indicate the *Xho*I linker). The PCR products were cloned into the *Kpn*I and *Xho*I sites of pGL3-Basic. The sequence and orientation of the insert were confirmed by sequencing. To express the ACVR2A protein, the coding region of *ACVR2A* (NM_007396, 664–2,205 bp) was PCR amplified from cDNA from LβT2 cells using a forward primer (5′-GCTCTAGAatgggagctgctgcaaagttggc-3′; uppercase letters indicate the *Xha*I linker) and a reverse primer (5′-CGGAATTCctaagcgtaatcaggaacgtcgtaagggtatagactagattctttgggaggaaagtc-3′; uppercase letters indicate the *Eco*RI linker, and underlined letters indicate the C-terminal hemagglutinin [HA] tag for ACVR2A, respectively). The PCR product was cloned into the *Xha*I and *EcoR*I sites of the pCAGGS vector [53]. The sequence and orientation of the insert were confirmed by sequencing. The expression of ACVR2A was confirmed by western blotting with an anti-HA antibody 3 F10 (Roche, Cat. #11 867 423 001, 100 ng/ml). Immunoreactivity was detected with a horseradish peroxidase-conjugated donkey anti-rat IgG antibody (Jackson ImmunoResearch, Cat. #712-035-153) and the ECL Prime Western Blotting Detection Reagent (GE Healthcare, Cat. #RPN2232). Chemiluminescence was detected with an ImageQuant LAS 4000 (GE Healthcare) and quantified using the ImageQuant TL Analysis Toolbox. LβT2 cells [33, 34] were maintained in DMEM with 10 % charcoal-stripped FCS (Gibco, Cat. #12676-029) supplemented with 2 mM l-glutamine, 20 nM dexamethasone (Sigma, Cat. #D4902), 0.1 mM non-essential amino acids (Gibco, Cat. #11140-050), and 100 units/ml penicillin and 100 μg/ml streptomycin.

To determine whether the expression level of *ACVR2A* affected *FSHB* reporter activity in response to activin A, we transfected 2 × 10^5^ cells per well in a 24-well plate with a mixture of 200 ng of the *FSHB* promoter-reporter vector, pCAGGS-*ACVR2A* (using the amounts of plasmid indicated in Fig. 3) and 10 ng of pRL-TK Renilla to determine transfection efficiencies. After 18 h, 50 ng/ml recombinant activin A (R&D, Cat. #338-AC; 50 μg/ml stock in 0.1 % bovine serum albumin/phosphate-buffered saline) was added to the culture medium, and luciferase assays were performed at 6 h post-activin A stimulation using the Dual Luciferase Reporter Assay System and the GloMax.

To examine co-transfection efficiencies in LβT2 cells, we used the pCAGGS-EGFP (Clontech, Cat. #6085-1) and pCAGGS-mCherry (Clontech, Cat. #632522) vectors. At 24 h post-transfection, cells were examined with a confocal microscope (FV1000, Olympus Optical) and fluorescence-positive cells were counted using ImageJ software, version 1.46 [54].

### Replication study

For the replication study, we used 1,433 samples from the remainder of the cohort from the same farm selected for the GWAS (Additional file 7). The g.48476925C > T SNP was genotyped by directly sequencing PCR products using a forward primer (5′-acaatctcctcgcgctcac-3′) and a reverse primer (5′-caagttctggtccaggctct-3′). PCR products were sequenced using the reverse primer and the BigDye Terminator v.3.1 Cycle Sequencing Kit (Applied Biosystems), followed by electrophoresis using an ABI 3730 sequencer (Applied Biosystems) and genotyping using SeqScape software, V2.5 (Applied Biosystems). The 3-bp indel (g.48476943_48476946insGGC) was PCR-amplified using a forward primer (5′-acaatctcctcgcgctcac-3′) and a reverse primer (5′-fluorescein amidite-caagttctggtccaggctct-3′). PCR products were electrophoresed using an ABI 3730 sequencer (Applied Biosystems) and genotyped using GeneScan analysis software (Applied Biosystems) and GeneMapper software v3.7 (Applied Biosystems).

### Estimation of the genetic variance explained by the haplotype and effect size of haplotype 

The effects of the haplotype were estimated as the least square means in generalized linear model (GLM) analyses. The statistical model for GLM analysis included fixed variables, such as the farm, birth year, and haplotype. The genetic variance explained by the haplotype was calculated based on estimates of the haplotype effect and the haplotype frequency [55]. Total genetic variance was estimated using the MTDF-REML program. The effect size of haplotype was estimated as the proportion of genetic variance explained by the haplotype.

## Availability of supporting data

The data sets supporting the results of this article are included within the article and its additional file.

## Additional files

Additional file 1:
**Distribution of ANAI**_4_ **in 10,399 cows in this study.** (PDF 31 kb) Additional file 2:
**Quantile-quantile plots of the GWAS results for ANAI**_4_ **.** The red dots represent the observed − log10 *P* values, and the straight line represents the expected − log10 *P* values under the null hypothesis. (PPTX 68 kb) Additional file 3:
**Genes on BTA2 in the ANAI**_4_ **-associated region.** The positions are based on the UMD3.1 assembly of the bovine genome. (XLSX 36 kb) Additional file 4:
**Relative expression of** ***ACVR2*** **in cow tissues and cells.** Relative *ACVR2A* expression levels in tissues and cells are indicated on the Y-axis. Total RNA was extracted from tissues (1–16), primary dermal fibroblasts (17) from 2 female Japanese Black cattle, or from bovine primary endometrial epithelial cells (18). Relative gene expression levels in the different tissues are shown as mean quantities relative to the value observed in ovarian tissue (dotted line). (PPTX 50 kb) Additional file 5:
**Transfection and co-transfection efficiencies of LβT2 cells.** (A) To examine the transfection efficiency of LβT2 cells, we used the pCAGGS-EGFP and pCAGGS-mCherry vectors. At 24 h post-transfection, double fluorescence-positive cells were counted using ImageJ software. The data shown represent the transfection efficiencies of LβT2 cells in 5 experiments. The average transfection efficiency was 8.8 ± 1.62 % (3,368 cells). (B–D) To examine co-transfection efficiency in LβT2 cells, we used the pCAGGS-EGFP and pCAGGS-mCherry vectors. At 24 h post-transfection, double fluorescence-positive cells (B, magenta) were counted using ImageJ software. All GFP positive cells (C, green) were mCherry-positive (D, red), representing 100 % of 3,368 cells. (PPTX 6646 kb) Additional file 6:
**Expression of pCAGGS-** ***ACVR2A*** **in LβT2 cells.** (A) To determine whether the pCAGGS-*ACVR2A* plasmid was expressed in LβT2 cells, we transfected 2 × 10^5^ cells per well in a 24-well plate with a mixture of 200 ng of the *FSHB* promoter-reporter plasmid, pCAGGS-*ACVR2A* (the amount of each plasmid is indicated in the lanes) and 10 ng of pRL-TK Renilla. HA-ACVR2A expression was confirmed by western blot analysis using an anti-HA antibody. Unstained Precision Plus protein standards were used as a marker (BioRad, Cat. #161-0363). HA-ACVR2A expression was detected with multiple bands (~57.8 kDa) because TGF-beta type-II receptors are normally modified by phosphorylation and glycosylation, causing then to migrate heterogeneously in sodium dodecyl sulfate-polyacrylamide gel electrophoresis gels. (B) Relative ACVR2A band intensities were measured using the ImageQuant TL Analysis Toolbox. The amounts of loaded proteins were calibrated by Coomassie Brilliant Blue-stained bands between 37 and 75 kDa. The bars represent the mean ± SEM observed in triplicate from 3 independent experiments. (PPTX 706 kb) Additional file 7:
**Distribution of ANAI**_4_ **in 1,433 cows for the replication study.** A sample population (*n* = 1,433) was derived from the remainder of the cohort from the same farm used for the GWAS. (PPTX 47 kb)

## Abbreviations

ANAI_4_: Average of the inverse of the number of artificially inseminations required for conception from first through fourth parity
BTA: Bovine (*Bos taurus*) chromosome
GWAS: Genome-wide association study
LD: Linkage disequilibrium
PCR: Polymerase chain reaction
QTL: Quantitative trait locus or loci
SNP: Single nucleotide polymorphism
