# Supplementary figures and images for: Genetic variants in the upstream region of activin receptor IIA are associated with female fertility in Japanese Black cattle
Source: BMC Genet. 2015 Oct 20;16:123. doi: 10.1186/s12863-015-0282-0 (PMC4618343; doi:10.1186/s12863-015-0282-0)

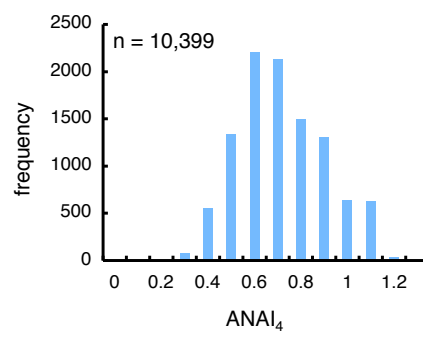

Supplement: Additional file 1: — Distribution of ANAI 4 in 10,399 cows in this study. (PDF 31 kb) [file 12863_2015_282_MOESM1_ESM.pdf]

## Slide 1
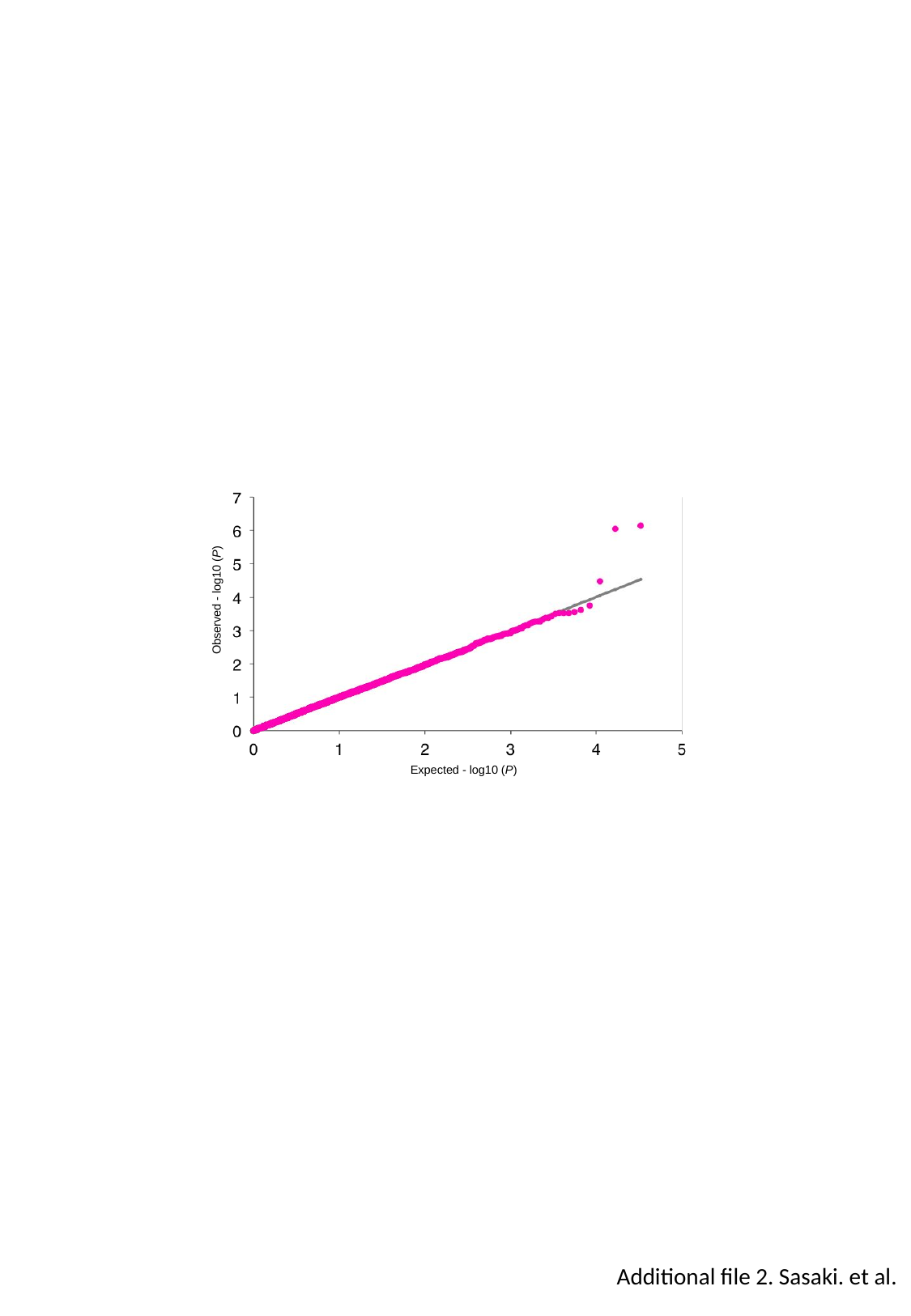

Observed - log10 (P)
Expected - log10 (P)
Additional file 2. Sasaki. et al.

Supplement: Additional file 2: — Quantile-quantile plots of the GWAS results for ANAI 4 . The red dots represent the observed − log10 P values, and the straight line represents the expected − log10 P values under the null hypothesis. (PPTX 68 kb) [file 12863_2015_282_MOESM2_ESM.pptx]

## Slide 1
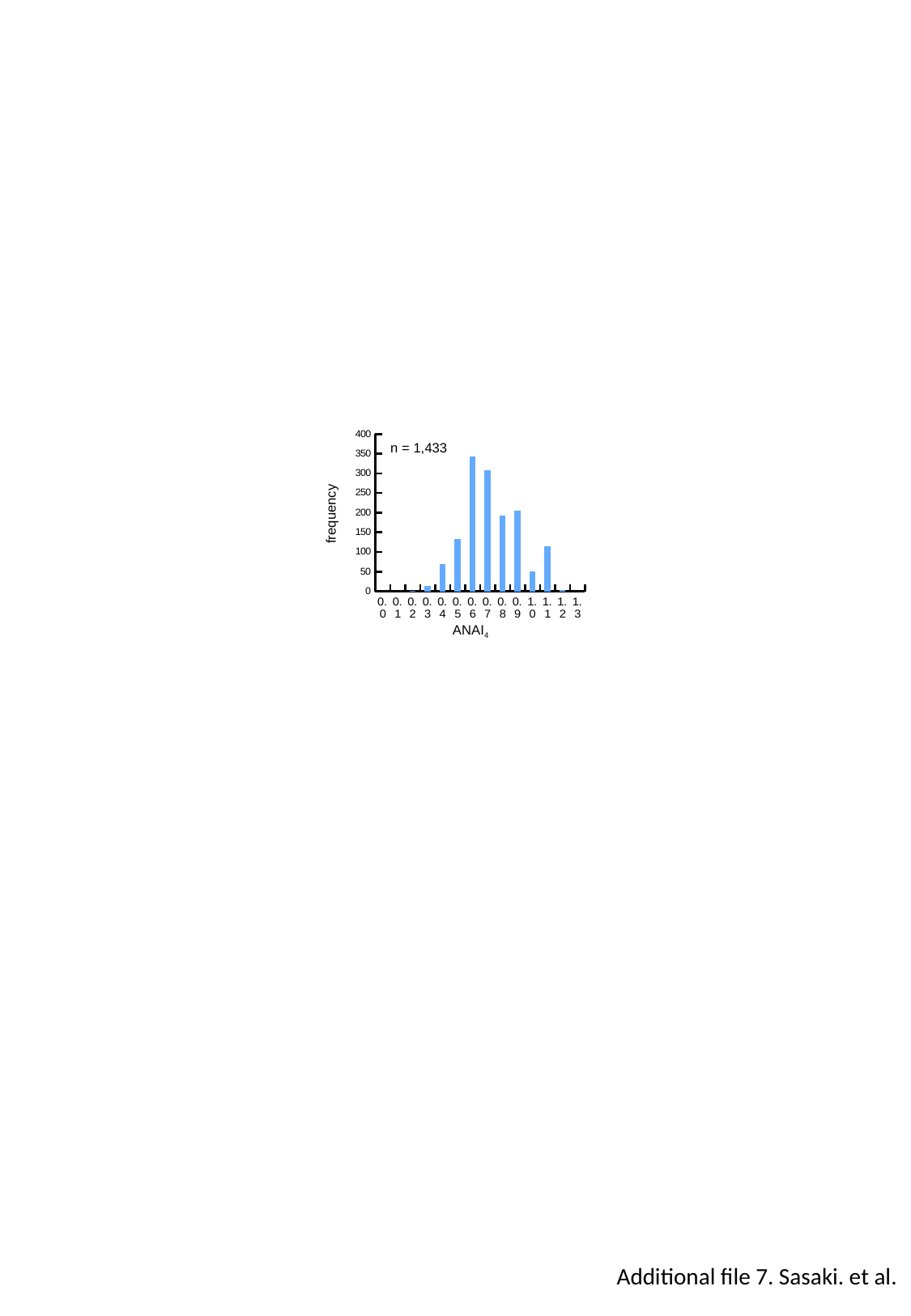

### Chart
| Category | |
|---|---|
| 0.0 | 0.0 |
| 0.1 | 0.0 |
| 0.2 | 1.0 |
| 0.3 | 14.0 |
| 0.4 | 70.0 |
| 0.5 | 133.0 |
| 0.6 | 342.0 |
| 0.7 | 307.0 |
| 0.8 | 192.0 |
| 0.9 | 205.0 |
| 1.0 | 51.0 |
| 1.1 | 115.0 |
| 1.2 | 3.0 |
| 1.3 | 0.0 |n = 1,433
frequency
ANAI4
Additional file 7. Sasaki. et al.

Supplement: Additional file 7: — Distribution of ANAI 4 in 1,433 cows for the replication study. A sample population (n = 1,433) was derived from the remainder of the cohort from the same farm used for the GWAS. (PPTX 47 kb) [file 12863_2015_282_MOESM7_ESM.pptx]
