# Supplementary material for: Genetic variants in the upstream region of activin receptor IIA are associated with female fertility in Japanese Black cattle
Source: BMC Genet. 2015 Oct 20;16:123. doi: 10.1186/s12863-015-0282-0 (PMC4618343; doi:10.1186/s12863-015-0282-0)
Supplement: Additional file 4: — Relative expression of ACVR2 in cow tissues and cells. Relative ACVR2A expression levels in tissues and cells are indicated on the Y-axis. Total RNA was extracted from tissues (1–16), primary dermal fibroblasts (17) from 2 female Japanese Black cattle, or from bovine primary endometrial epithelial cells (18). Relative gene expression levels in the different tissues are shown as mean quantities relative to the value observed in ovarian tissue (dotted line). (PPTX 50 kb) [file 12863_2015_282_MOESM4_ESM.pptx]

## Slide 1
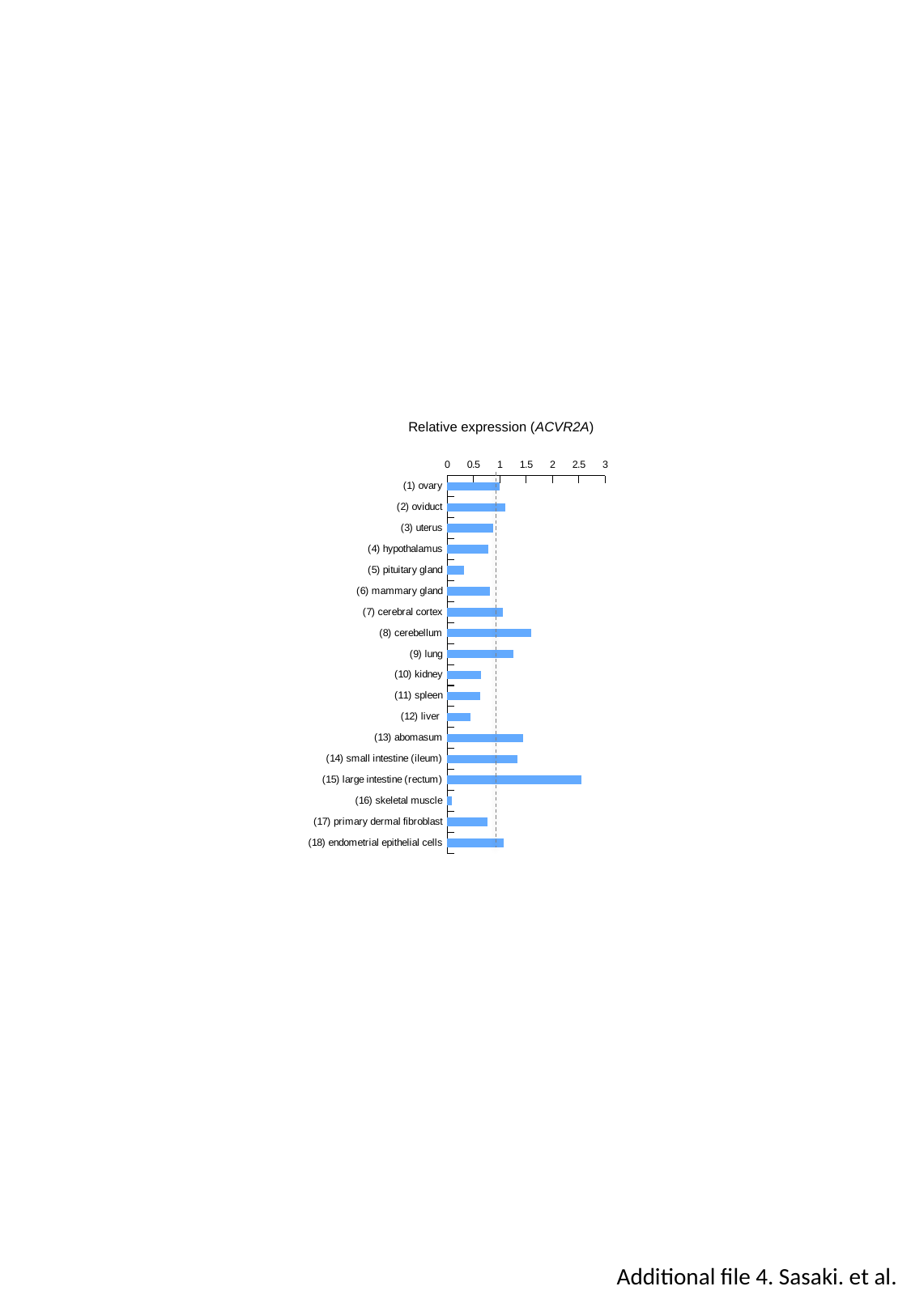

Relative expression (ACVR2A)
### Chart
| Category | relative expression |
|---|---|
| (1) ovary | 1.0 |
| (2) oviduct | 1.1056627 |
| (3) uterus | 0.87284493 |
| (4) hypothalamus | 0.7882509 |
| (5) pituitary gland | 0.324237 |
| (6) mammary gland | 0.81429464 |
| (7) cerebral cortex | 1.0653657 |
| (8) cerebellum | 1.5929981 |
| (9) lung | 1.2602025 |
| (10) kidney | 0.63897663 |
| (11) spleen | 0.62222815 |
| (12) liver | 0.44510487 |
| (13) abomasum | 1.4488208 |
| (14) small intestine (ileum) | 1.3294463 |
| (15) large intestine (rectum) | 2.5497947 |
| (16) skeletal muscle | 0.091613434 |
| (17) primary dermal fibroblast | 0.773053 |
| (18) endometrial epithelial cells | 1.0753398 |Additional file 4. Sasaki. et al.
