# Supplementary material for: Genetic variants in the upstream region of activin receptor IIA are associated with female fertility in Japanese Black cattle
Source: BMC Genet. 2015 Oct 20;16:123. doi: 10.1186/s12863-015-0282-0 (PMC4618343; doi:10.1186/s12863-015-0282-0)
Supplement: Additional file 5: — Transfection and co-transfection efficiencies of LβT2 cells. (A) To examine the transfection efficiency of LβT2 cells, we used the pCAGGS-EGFP and pCAGGS-mCherry vectors. At 24 h post-transfection, double fluorescence-positive cells were counted using ImageJ software. The data shown represent the transfection efficiencies of LβT2 cells in 5 experiments. The average transfection efficiency was 8.8 ± 1.62 % (3,368 cells). (B–D) To examine co-transfection efficiency in LβT2 cells, we used the pCAGGS-EGFP and pCAGGS-mCherry vectors. At 24 h post-transfection, double fluorescence-positive cells (B, magenta) were counted using ImageJ software. All GFP positive cells (C, green) were mCherry-positive (D, red), representing 100 % of 3,368 cells. (PPTX 6646 kb) [file 12863_2015_282_MOESM5_ESM.pptx]

## Slide 1
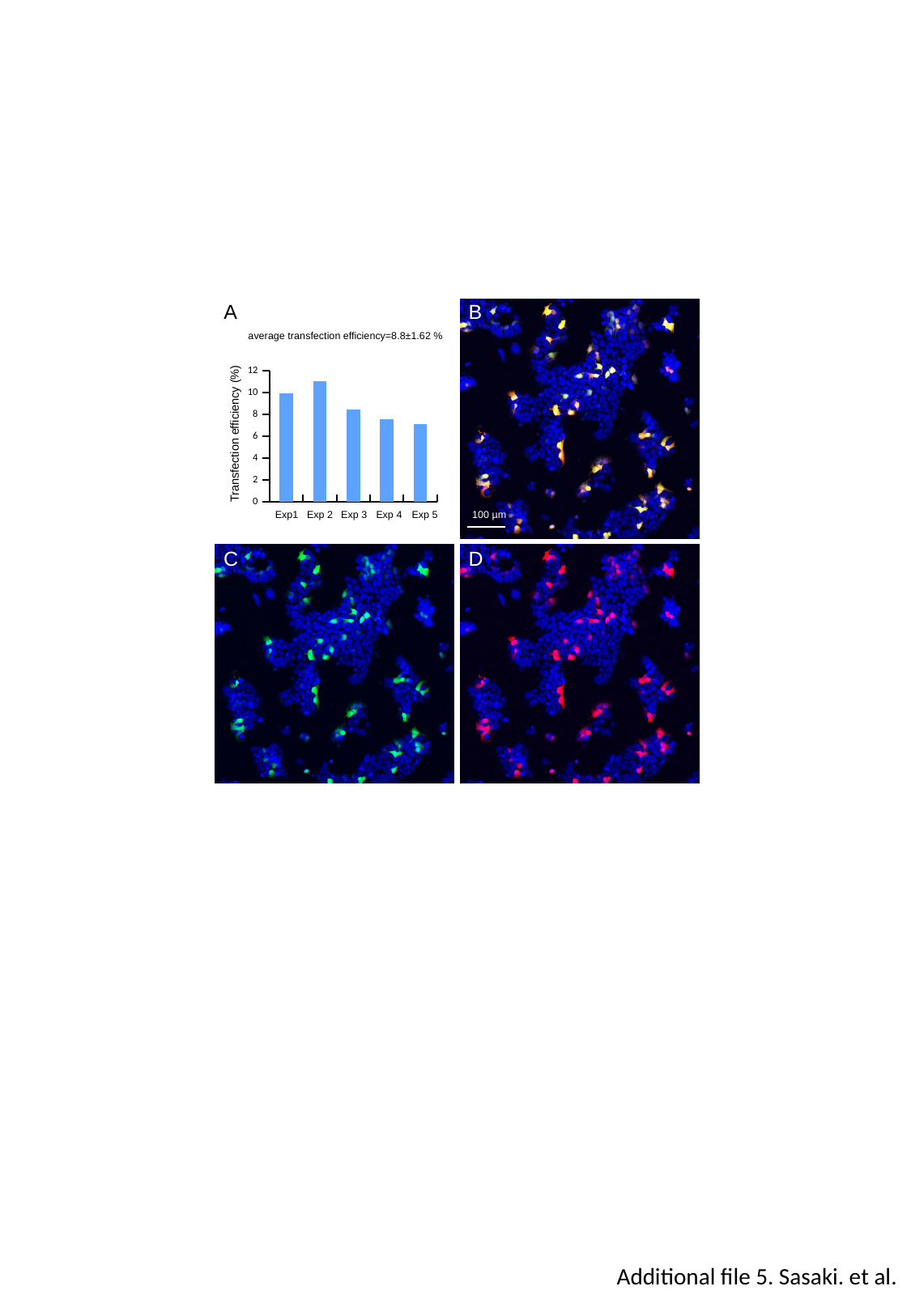

A
B
average transfection efficiency=8.8±1.62 %
### Chart
| Category | transfection efficiency(%) |
|---|---|
| Experiment_1 | 9.888059701492498 |
| Experiment_2 | 11.00671140939597 |
| Experiment_3 | 8.46645367412141 |
| Experiment_4 | 7.536231884057971 |
| Experiment_5 | 7.133592736705577 |Transfection efficiency (%)
Exp1
Exp 2
Exp 3
Exp 4
Exp 5
100 µm
C
D
Additional file 5. Sasaki. et al.
