# Supplementary material for: Genetic variants in the upstream region of activin receptor IIA are associated with female fertility in Japanese Black cattle
Source: BMC Genet. 2015 Oct 20;16:123. doi: 10.1186/s12863-015-0282-0 (PMC4618343; doi:10.1186/s12863-015-0282-0)
Supplement: Additional file 6: — Expression of pCAGGS- ACVR2A in LβT2 cells. (A) To determine whether the pCAGGS-ACVR2A plasmid was expressed in LβT2 cells, we transfected 2 × 10^5^ cells per well in a 24-well plate with a mixture of 200 ng of the FSHB promoter-reporter plasmid, pCAGGS-ACVR2A (the amount of each plasmid is indicated in the lanes) and 10 ng of pRL-TK Renilla. HA-ACVR2A expression was confirmed by western blot analysis using an anti-HA antibody. Unstained Precision Plus protein standards were used as a marker (BioRad, Cat. #161-0363). HA-ACVR2A expression was detected with multiple bands (~57.8 kDa) because TGF-beta type-II receptors are normally modified by phosphorylation and glycosylation, causing then to migrate heterogeneously in sodium dodecyl sulfate-polyacrylamide gel electrophoresis gels. (B) Relative ACVR2A band intensities were measured using the ImageQuant TL Analysis Toolbox. The amounts of loaded proteins were calibrated by Coomassie Brilliant Blue-stained bands between 37 and 75 kDa. The bars represent the mean ± SEM observed in triplicate from 3 independent experiments. (PPTX 706 kb) [file 12863_2015_282_MOESM6_ESM.pptx]

## Slide 1
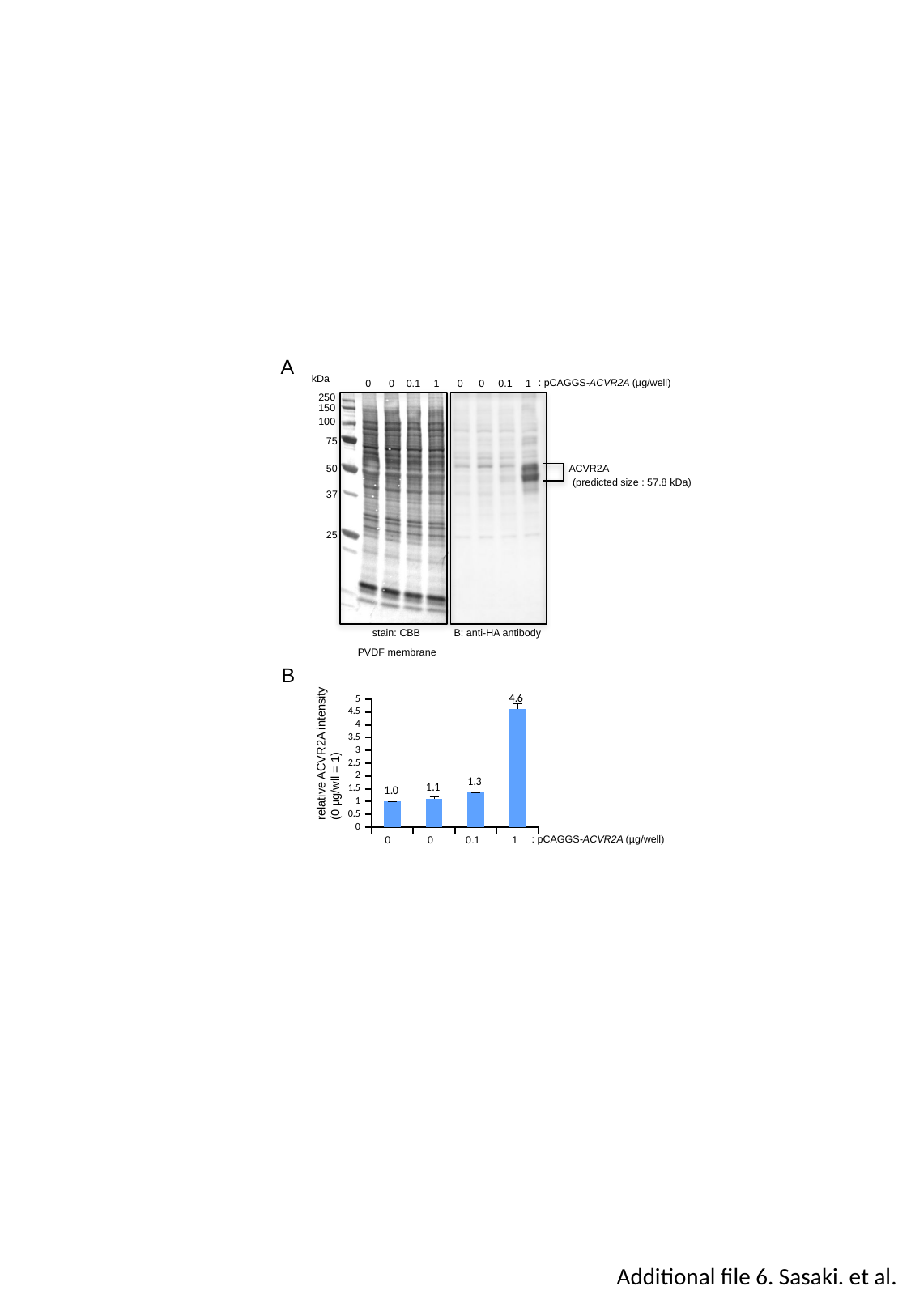

A
kDa
: pCAGGS-ACVR2A (µg/well)
0
0
0.1
1
0
0
0.1
1
250
150
100
75
50
ACVR2A
 (predicted size : 57.8 kDa)
37
25
stain: CBB
B: anti-HA antibody
PVDF membrane
B
### Chart
| Category | average |
|---|---|
| 0µg/wll | 1.0 |
| 0µg/wll | 1.117939607857215 |
| 0.1µg/wll | 1.348471365750915 |
| 1µg/wll | 4.613570542015333 |relative ACVR2A intensity
(0 µg/wll = 1)
: pCAGGS-ACVR2A (µg/well)
0
0
0.1
1
Additional file 6. Sasaki. et al.
